# Oral Health Care in the United States

**DOI:** 10.3390/dj14050265

**Published:** 2026-05-02

**Authors:** Duangporn Duangthip, Sherif Ammar, Frederick Howard, Xi Chen

**Affiliations:** 1 Division of Dental Public Health, College of Dentistry, The Ohio State University, Columbus, OH 43210, USA; howard.2078@osu.edu (F.H.); chen.13047@osu.edu (X.C.); 2 College of Public Health, The Ohio State University, Columbus, OH 43210, USA; ammar.23@osu.edu

**Keywords:** oral health, dental caries, oral disease, periodontal diseases, workforce, healthcare, public health

## Abstract

An updated understanding of the U.S. oral health care system is essential for addressing the burden of oral disease, high dental expenditures, and persistent inequities in access. This narrative review synthesizes current evidence on the prevalence of major oral diseases, dental care delivery, financing, dental workforce, and public health initiatives, and highlights the challenges and future opportunities in the U.S. A comprehensive search of PubMed, Google Scholar, and reports from U.S. federal agencies and professional organizations was conducted between September 2025 and March 2026. Following the latest National Health and Nutrition Examination Survey, untreated caries remains widespread, affecting 11% of children (ages 2–5), 10% of adolescents (ages 12–19), 21% of adults (ages 35–49), and 12% of older adults (ages 65–74). Periodontal diseases are common, with 42% of adults aged 30 years or older having periodontitis. Oral cancer incidence stands at 11.5 per 100,000 and increases sharply with advancing age. Edentulism among older adults (ages 65–74) was approximately 11%. The U.S. dental workforce includes over 200,000 dentists, yet shortages affect rural and low-income areas, with 62 million Americans living in Dental Health Professional Shortage Areas. Dental care is primarily delivered through private practices, supplemented by community health centers. Financing relies mostly on private insurance and out-of-pocket payments, while the coverage of public programs like Medicaid varies across states, and Medicare generally excludes routine dental care for older adults. Water fluoridation remains widespread, yet ongoing debates highlight persistent challenges. School-based dental sealants and topical fluoride programs are widely recognized as cost-effective and scalable, offering substantial benefits at the population level. Nevertheless, community-based preventive measures are often hindered by resource constraints, inequitable access, and in some cases political conflicts. In summary, oral diseases remain prevalent in the U.S. Limited public coverage, workforce shortages in rural or underserved areas, and uneven access to dental care highlight the need for systemic reforms to improve oral health equity. These findings point to the importance of strengthening dental public health research and coordinated policy action to reduce structural barriers and expand access to dental care.

## 1. Introduction

The United States of America, or the United States (U.S.), is a federal republic located in North America, consisting of 50 states, the District of Columbia, and several territories. It covers a total land area of about 9.15 [1] million square kilometers, making it the third-largest country in the world by both area and population. The U.S. has a highly developed economy and ranks among the highest globally in terms of gross domestic product (GDP), innovation, and scientific research. In 2024, the per capita GDP of the U.S. was approximately US $85,810 [1], and the total expenditure on health was about 17.6.% of GDP, one of the highest in the world [2]. The population of the U.S. was about 340 million in 2024 [3]. Like many developed countries, population aging is a growing concern [4]. The proportion of elderly people aged 65 years or older reached 17.3% in 2022 and is projected to increase to 22.0% by 2040 [5]. The median age of the U.S. population was 39.1 years in 2024 [5]. Life expectancy at birth in 2023 was 75.8 years for males and 81.1 years for females [6]. In terms of the health workforce, the U.S. has approximately 1.1 million active physicians, with a doctor-to-population ratio of about 1:313 [7], while the number of active or registered dentists was 202,485, with a dentist-to-population ratio of about 1:1700 [8]. The estimated global economic impact of five main oral conditions (dental caries of primary teeth, dental caries of permanent teeth, periodontitis, edentulism, and others) was approximately US $710 billion. Among all the countries included, the U.S. recorded the highest productivity losses (211.98 billion), with direct costs of $133.51 billion and indirect costs of $78.47 billion—the largest in the world [9]. To address the high expenditure on major dental problems, evidence points to a combination of preventive strategies, policy reforms, and innovative care models. Updating oral health care in the U.S. is critical to addressing the persistent burden of oral diseases and inequities in access.

This narrative review aims to synthesize current evidence on the prevalence of major oral diseases, dental care delivery, financing, dental workforce, and public health initiatives, and highlight the challenges and future opportunities for improving oral health in the U.S. We adopted the narrative synthesis by summarizing findings across studies, integrating diverse evidence, and providing an overview of key themes and patterns. This review is organized into six thematic sections, including (1) oral health status, (2) oral health care system, (3) financing of dental care, (4) dental workforce and education, (5) dental public health program and preventive care, (6) challenges and opportunities. The details of each thematic area are described in the next sections. By synthesizing evidence from diverse sources into a coherent overview, the review equips clinicians, policymakers, and researchers with insights to address persistent disparities and gaps in coverage. Beyond the U.S. context, the analysis offers valuable guidance for countries facing similar challenges in the oral health care system. The discussion on community-based strategies provides transferable lessons for global health systems seeking to strengthen oral health equity. Thus, the review not only informs U.S. stakeholders but also offers international scholars a deeper understanding of the U.S. oral health care landscape, fostering global dialog within the global public health community.

## 2. Methods

Although narrative in design, the review incorporated systematic elements to minimize selection bias and enhance reproducibility. A comprehensive search was conducted across major biomedical and public health databases, including PubMed/MEDLINE, Scopus, Web of Science, and Google Scholar, as well as federal sources such as the Centers for Disease Control and Prevention (CDC), and the Health Resources and Services Administration (HRSA). The search covered the literature published from January 2000 through March 2026, with particular emphasis on the most recent decade to ensure the currency of evidence.

Search strategies combined controlled vocabulary, such as MeSH terms, with free-text keywords. The search included terms such as “oral health care system”, “United States” or “U.S.”, “dental care delivery”, “dental workforce”, “dental education”, “oral health financing”, “public health dentistry”, “oral health policy”, and “national oral health survey”. Boolean operators (AND/OR) were used to refine and expand the search as appropriate. Studies were excluded if they examined countries outside the United States, lacked empirical or policy-relevant content, consisted solely of opinion or commentary, or represented duplicate or outdated versions of previously published reports.

## 3. Oral Health Status

Three major dental diseases, including dental caries, periodontal diseases, and oral cancer are reviewed in this paper.

### 3.1. Dental Caries

Data on dental caries status, number of retained teeth, and edentulism from the most recent National Health and Nutrition Examination Survey (NHANES) 2017–2020 [10,11] reported by CDC are summarized in Table 1. For comparison, findings from the previous NHANES 2011–2016 are presented in Table 2 [12].

Preschool children (ages 2–5 years)

Based on NHANES 2017–2020 data, approximately 11.1% of children aged 2–5 years had untreated decay in their primary teeth, with a mean of 1.8 decayed and 2.6 filled teeth [10]. Compared with NHANES 2011–2016, the overall prevalence of dental caries in this age group has remained relatively stable. In 2011–2016, the prevalence of untreated decay was 10.4%, with mean values of 1.6 decayed and 2.8 filled teeth [12].

School children (ages 6–11 years)

The prevalence of untreated decay in permanent teeth was approximately 2.6%, with a mean of 0.3 decayed and 1.6 filled teeth [10]. Compared with NHANES 2011–2016, the prevalence of untreated dental caries in this age group declined slightly, from 5.2% in 2011–2016 to 2.6% in 2017–2020. Similarly, the mean number of decayed permanent teeth (DT) decreased from 0.5 to 0.3, whereas the mean number of filled teeth (FT) remained unchanged (1.6) [10,12].

Adolescents (ages 12–19 years)

Among adolescents, 10.4% had at least one untreated cavity in their permanent teeth, with a mean of 0.4 decayed and 3.7 filled teeth [10]. Compared with NHANES 2011–2016, untreated decay declined from 16.6% to 10.4%, and the mean decayed teeth also decreased from 0.7 to 0.4, whereas the mean filled teeth increased slightly from 3.4 to 3.7 [10,12].

Working-age adults (ages 20–64 years)

Oral health among adults has improved over time between NHANES 2011–2016 and 2017–2020. Both the prevalence of untreated decay and the mean number of decayed and filled teeth decreased across working-age adults. Specifically, untreated decay among young adults (ages 20–34) declined from 29.3% to 21.8%. In the middle-aged group (ages 35–49), prevalence decreased from 26.4% to 21.4%. A similar decline was also observed among adults aged 50–64, with untreated caries prevalence dropping from 21.5% to 17.3%. However, the mean number of retained teeth remained stable at approximately 23 teeth in both survey periods [10,12].

Older adults (ages 65 and older)

Between NHANES 2011–2016 and 2017–2020, untreated decay decreased and edentulism declined among older adults, while tooth retention remained stable or slightly improved [10,12]. Specifically, the prevalence of untreated decay declined among adults aged 65–74 from 15.4% to 12.4% and among those aged 75 years and older from 16.5% to 12.6%. The mean number of decayed teeth decreased in both groups (from 0.3 to 0.2). Mean filled teeth declined slightly among adults aged 65 to 74 years (from 9.9 to 9.0) but remained unchanged among those aged 75 years or older (from 9.6 to 9.7). The mean number of retained teeth (21.7) was stable for adults aged 65 to 74 years and increased slightly among those aged 75 and older (from 19.5 to 19.8). Edentulism also declined in both age groups from 13.0% to 11.4% among adults aged 65 to 74 and from 22.5% to 19.7% among those aged 75 years and older.

### 3.2. Periodontal Disease

The latest national statistics on periodontal disease in the United States come from the NHANES study conducted between 2009 and 2014 [13]. These statistics indicate that approximately 42% of adults aged 30 and older have some form of periodontitis, with nearly 8% suffering from severe cases. However, these findings are now over ten years old, and no new data has been released since then. The United States has not conducted a nationally representative periodontal examination since NHANES 2009–2014. This may be because periodontal charting is resource-intensive, requiring trained and calibrated examiners, substantial chairside time, and costly clinical infrastructure. Persistent funding constraints and operational pressures within NHANES have further limited the feasibility of continuing labor-intensive clinical assessments, while federal surveillance priorities have shifted toward other public health areas, reducing emphasis on oral health monitoring. This lack of updated information makes it challenging to assess whether the prevalence of periodontal disease has changed recently. Given trends in oral health, an aging population, and improvements in preventive care, there is a need for updated national data to accurately understand the current state of periodontal disease [14,15].

Recent evidence from a retrospective analysis of electronic health records in an academic dental setting suggests that the burden may be higher than previously reported: more than half (55.8%) of patients had periodontitis, with the majority of cases being severe, and severity was strongly associated with older age and male sex [16]. A substantial body of research has also reinforced the link between periodontal disease and systemic conditions, including cardiovascular disease [17], diabetes [18], and dementia [19], underscoring the importance of updated epidemiologic data to guide prevention and management strategies.

A recent study used Global Burden of Disease 2021 data (1990–2021) and applied joinpoint regression, decomposition analysis, and a Bayesian age–period–cohort model to assess and project severe periodontitis trends in the U.S. through 2050 [20]. The U.S. has a lower burden of severe periodontitis than global levels, likely reflecting long-standing preventive care practices. However, notable age and sex differences highlight the need for targeted strategies for men and adults aged 50–64 years with notable regional variation—New York and North Carolina showed the highest incidence, while Nevada and Arizona had the fastest growth. Significant regional disparities further underscore the importance of equitable resource allocation across states. Finally, population growth and aging continue to challenge efforts to reduce periodontal disease burden nationally and globally.

### 3.3. Oral and Pharyngeal Cancers

Oral cancer remains a significant public health concern. Based on the most available updated data from the Surveillance, Epidemiology, and End Results program for 2015–2019 [21], the overall age-adjusted incidence of oral cavity and pharyngeal cancers was 11.5 per 100,000, increasing sharply with advanced age—from 1.4 per 100,000 among individuals aged 15–39 years to 43.7 per 100,000 among those aged 65–74 years (Table 3). Tobacco-related cancers have declined due to the decline in smoking, but human papillomavirus (HPV)-related oropharyngeal cancers have risen substantially [22].

In summary, the NHANES data highlight important trends in oral health across the lifespan. Among preschool children, the prevalence of untreated decay in primary teeth has remained relatively stable, while school-aged children experienced modest declines in untreated decay in permanent teeth. Adolescents showed more notable improvements, with fewer untreated decay. Working-age adults demonstrated consistent progress, with reductions in untreated decay across all subgroups, although the average number of retained teeth remained unchanged. Older adults also benefited, showing declines in untreated decay and edentulism. In contrast, national statistics on periodontal disease remain outdated, with the last estimates from 2009–2014 indicating that nearly half of adults aged 30 and older were affected, underscoring the need for updated surveillance. Oral and pharyngeal cancers continue to pose a major public health challenge, with incidence rising sharply with age and shifting patterns linked to HPV-related disease.

## 4. Oral Health Care System

Dental care in the U.S. is delivered through a complex system of private, public, and non-governmental organization providers that significantly vary in terms of accessibility and workforce composition [8]. Private practices remain the predominant setting in which dental care is delivered. Most dentists who serve insured and self-paying patients operate either individual practices or Dental Service Organizations, corporate offices that share administrative costs. While data as recent as 2023 shows that 73% of working dentists are practice owners, this is down from 85% in 2005, showing a significant trend toward group offices [24]. These settings offer quality service and greater patient choice but often exclude low-income and uninsured populations, reinforcing long-standing inequities in access [25]. Privately operated dental offices that accept Medicaid tend to offer comparatively fewer services [26].

Public programs play a role for specific populations, but access varies significantly across states. Community Health Centers (CHCs), including Federally Qualified Health Centers, represent a critical safety-net for populations not well served by private practice [27]. CHCs primarily serve socioeconomically disadvantaged populations and increasingly care for patients in rural areas. These patients are often uninsured or enrolled in government programs such as Medicaid, which covers low-income individuals, and Medicare, which serves adults over 65 and those with certain disabilities. Additionally, CHCs often serve patients with substantial chronic disease burdens or complex health needs, including the elderly who are in poor health [28]. While these centers significantly expand access, they face persistent challenges including workforce shortages, chronic underfunding, and inconsistent ability to meet growing demand [29].

Dental schools and university-based clinics play a significant role in the U.S. oral health care system. In 2023, 67 accredited dental schools operated clinics that provided much-needed care to local communities, supervised by specialists [30]. These teaching clinics combine clinical training with patient care, enabling students to gain practical experience under faculty guidance while delivering high-quality dental services. Dental school clinics disproportionately treat publicly insured and underserved populations; in 2022, 37% of patients were covered by Medicaid or Children’s Health Insurance Program (CHIP) [30]. Because the clinics are structured around training and education, clinical capacity can be limited, and appointment times can be longer than in private practices [31].

Hospital-based dental clinics are a relatively uncommon and under-documented venue for care in the United States. They primarily treat patients with significant medical needs, including children requiring general anesthesia, individuals with cancer or maxillofacial irregularities, geriatrics, and those presenting with dental emergencies [32]. Many hospital-based dental clinics are tied to dental schools or teaching hospitals. They provide advanced specialty training such as oral surgery and pediatric dentistry. U.S. hospital dental clinics act as specialty hubs for complex cases and training, rather than providing general dental care for the public. Medicaid expansion has increased hospital-based emergency department dental visits, underscoring the need for expanded hospital-based services [32]. Medical–dental integration is the effort to bring oral health into mainstream healthcare so that medical and dental providers work together to deliver more coordinated, preventive, whole-person care. Dental hygienists are becoming central to expanding innovative, equity-focused oral health care models, especially as key leaders in advancing medical–dental integration [33]. Evidence showed that primary care providers can help close the medical–dental divide by incorporating basic preventive oral health services into routine medical visits [34]. Recently, there has been a growing trend of physician involvement in preventive dental care, particularly in early childhood settings. Physicians are increasingly administering fluoride treatments like sodium fluoride varnish and silver diamine fluoride in non-dental medical settings [35].

Mobile dental programs, often run by public health departments or nonprofit organizations, further extend care to rural communities, schools, older adults, and populations facing mobility barriers. Increasingly, for-profit mobile clinics provide dental services in corporate environments that aim to minimize the disruption of workforce productivity [36]. Although highly effective in increasing reach, mobile programs face logistical challenges, unstable funding, and limited ability for follow-up visits or continuity of care [37].

Geographic disparities in workforce composition and distribution shape access across all these models of dental care. Several strategies for care have emerged while attending to the needs of specific geographic populations, from urban centers to rural settings. Spatial analyses indicate that millions of U.S. residents live in “dental deserts”—areas with minimal or no local providers—resulting in delayed treatment, increased emergency department utilization, and widening inequities in oral disease burden [38,39]. CHCs and mobile programs partially mitigate these gaps but remain insufficient to fully address rural accessibility challenges.

Teledentistry is currently positioned as an adjunct to the existing oral health care system, using live video, store-and-forward data, remote monitoring, and mobile health tools to expand access to care [40]. Emerging evidence shows that synchronous models can help rural children and caregivers complete complex treatment needs [41], while asynchronous approaches can create a reliable dental home for long-term care residents and improve access to preventive and disease-management services for older adults [42]. Most available studies, however, emphasize feasibility or short-term outcomes. Stronger longitudinal research is needed to evaluate disease progression, continuity of care, sustained access gains, and the long-term effectiveness of teledentistry across diverse populations and care settings.

In the face of structural constraints that each of these models present, dental therapists have gained traction as a strategy to expand access in underserved communities. Research consistently shows that dental therapists provide safe, high-quality care, reduce wait times, and increase service availability in rural and low-income areas [43]. Evidence from Minnesota and tribal health systems demonstrates improvements in preventive service delivery and enhanced provider and patient satisfaction [44,45].

In sum, dental care in the United States is delivered through multiple models, including private practices, safety-net providers, and mobile programs. While these approaches offer varied options for dental care, differences in access related to geography, socioeconomic factors, and workforce distribution highlight opportunities for greater coordination to improve oral health outcomes nationwide.

## 5. Financing of Dental Care

Like overall health spending, dental expenditures in the United States have steadily increased over the past two decades. In 2023, American consumers spent $174 billion on dental care, a 2.5% increase from 2022 [46]. This long-term growth is largely driven by demographic changes, rising demand for restorative and specialty services, and increasing provider fees [47,48].

Although national dental expenditures continue to rise, the distribution of payment sources has remained relatively stable. About 40% of dental costs are paid out-of-pocket (Table 4), 35% through private dental insurance, and the remainder through public programs such as Medicaid and Medicare, which steadily increased in recent years [46]. This pattern is different from medical care, where private and public insurance cover a much larger share of costs [49]. Dentistry mostly operates under a fee-for-service payment model. As a result, patients often face high out-of-pocket expenses and must pay a large portion of their dental costs themselves, especially for major restorative, prosthodontic, and implant treatments [50]. Historically, public insurance has played only a limited role in dental coverage, which contributes to ongoing disparities in access to care and oral health outcomes across income and racial/ethnic groups [51]. The payment sources and share of dental expenditure are summarized in Table 4.

### 5.1. Private Dental Insurance and Out-of-Pocket Spending

Private dental insurance and out-of-pocket payments account for the majority of dental spending in the U.S. [46]. Most private dental insurance plans are employer-sponsored as part of employee benefit packages. For many working-age adults, private insurance is the primary way they obtain dental benefits in the U.S. [52]. These plans usually adopt a ‘preventive-centered’ model, in which diagnostic and preventive services are incentivized, while other treatments (e.g., major restorations and implant procedures) require higher cost-sharing or may be subject to waiting periods [52]. Despite inflation, annual maximum benefit limits of dental insurance plans, around $1000–1500, have remained virtually unchanged for decades, substantially diminishing the real value of coverage [53]. While this structure effectively promotes preventive and routine care, unlike medical insurance, dental insurance does not offer robust financial protection for high-cost treatments. As a result, out-of-pocket spending remains disproportionately high, even for insured individuals [48,50]. This is a particular concern for those seeking crowns, implants, orthodontic treatment, or full-mouth reconstructions. Studies have consistently identified cost as the primary barrier to accessing dental care. Many people, especially those with lower socioeconomic status, delay or forego recommended treatments due to financial concerns [54,55].

### 5.2. Medicaid Dental Benefits

Medicaid is the primary public insurance program for low-income individuals, including children, pregnant women, working-age adults, people with disabilities, and some older adults [56]. For children, comprehensive coverage of preventive and restorative services is federally mandated through the Early and Periodic Screening, Diagnostic, and Treatment program Centers for Medicare & Medicaid Services (CMS). However, adult dental benefits are optional and vary widely across states [56,57].

According to the Center for Health Care Strategies, among the 50 states and the District of Columbia, 4 states provided no adult dental benefits, 14 covered emergency-only services, 17 included limited benefits, and 17 extended comprehensive dental benefits for Medicaid populations in 2018 [58]. Dental benefits under state Medicaid programs typically fall into three categories: (1) emergency treatment to relieve pain, control bleeding, or address traumatic injury; (2) limited coverage, including diagnostic, preventive, and minor restorative procedures recognized by the American Dental Association (ADA); and (3) extensive coverage, including a comprehensive mix of ADA-approved diagnostic, preventive, and both minor and major restorative procedures [56]. In many states, even among those with extensive Medicaid adult dental benefits, dental services are subject to annual expenditure caps, although in recent years an increasing number of states have removed these limits [57].

### 5.3. Children’s Health Insurance Program (CHIP)

The CHIP complements Medicaid by extending public coverage to children in families with incomes that are too high for Medicaid but insufficient to afford private insurance [58]. In many states, CHIP offers comprehensive dental benefits with a strong emphasis on prevention. As of 2024, 37.6 million children, approximately half of all U.S. children, were enrolled in Medicaid or CHIP [59]. Benefiting from these public programs, children’s dental care utilization has improved substantially over the past two decades, as shown by increasing rates of preventive visits, fluoride treatments, and dental sealants [10,60,61]. However, low provider participation and ongoing workforce shortages in rural and underserved areas continue to constrain the capacity of these programs to improve equitable access to oral health care [62,63].

### 5.4. Medicare Dental Coverage

Medicare primarily covers adults aged 65 and older and certain younger adults with disabilities. Historically, Medicare covered only dental procedures that were essential to the clinical success of medical care (e.g., jaw fracture surgery or extraction of teeth prior to radiation therapy) and excluded routine dental services. Beginning in 2023, the CMS expanded Medicare’s definition of medically necessary dental services to include a broader set of clinical situations that are directly linked to major medical treatments [52]. Under these updated rules, Medicare can cover dental evaluations and treatment when they are required to reduce medical risk and support the success of a covered procedure (e.g., organ transplantation, cardiac valve repair or replacement, head and neck cancer therapy, certain neurosurgical procedures, and immunosuppressive therapy). In these circumstances, covered services may include exams, radiographs, extractions, treatment of acute infections, and other procedures needed to eliminate sources of oral disease that could interfere with the medical intervention [59]. Importantly, these dental treatments must be directly tied to the medical procedure and justified as necessary for its safe completion.

Despite this expansion, Medicare still does not provide a comprehensive routine dental benefit. Preventive services, restorative treatments (e.g., fillings, crowns, dentures, implants), and other routine care remain excluded unless they are specifically required to support a covered medical procedure [52]. In addition, although CMS has issued policy direction, the complexity of reimbursement processes, documentation requirements, and coverage criteria may create uncertainty for providers and lead to variation in implementation across regions. Many Medicare beneficiaries may have difficulty identifying dental providers who are willing to enroll and navigate Medicare billing requirements for medically necessary services [64]. Low-income and rural beneficiaries may face particular challenges in obtaining timely dental clearance prior to major medical procedures, which may delay care [64].

### 5.5. Impact of Payment Structure on Dental Utilization and Access

As discussed, the payment structure of U.S. dentistry is fragmented and heavily reliant on private payment sources. This financing model strongly influences patterns of dental care utilization and contributes directly to persistent disparities in access and treatment outcomes [65]. Individuals without insurance, or with limited benefits, often delay treatment until pain, infection, or other urgent situations arise. Adult beneficiaries in states with comprehensive Medicaid dental benefits utilize significantly more preventive and restorative services than those in states with limited or no benefits, highlighting the impact of policy design [57]. On the other hand, low Medicaid reimbursement rates and complex administrative requirements also contribute to limited dentist participation, appointment shortages, and long wait times, substantially restricting access to dental care in rural and low-income urban areas [25]. Children have benefited from improvements in Medicaid and CHIP dental coverage [12], yet racial, ethnic, and geographic disparities persist, especially among minority and rural populations [62]. Because Medicare excludes routine dental care and private insurance plans are limited in scope and dollar amount, older adults face high out-of-pocket costs. These financial barriers are associated with high rates of untreated caries, periodontal disease, edentulism, xerostomia, and related oral–systemic comorbidities [66,67,68].

## 6. Dental Workforce and Education

The U.S. has a large and diverse dental workforce, including general dentists, specialists, dental hygienists, and dental assistants. Dental hygienists play a critical role in preventive care, though their scope of practice varies by state. Dental education is offered in 79 accredited dental schools, with an emphasis on biomedical sciences, clinical skills, and increasingly, interprofessional education. Despite a relatively high dentist-to-population ratio overall, shortages remain in rural and underserved areas. A summary of dental degrees, number of schools or institutions, and number of registered dental professionals is displayed in Table 5.

The U.S. has one of the largest oral health workforces in the world. In 2024, there were 202,485 [8] practicing dentists, a ratio of about one for every 1700 people. Most dentists are general practitioners, though nearly one in five are specialists. Training requires completion of a four-year program leading to either a DDS or DMD degree, and as of September 2025 [71], there are 79 accredited dental schools in the U.S. For internationally trained dentists, licensure typically requires completing a U.S. program, though some states allow licensing after a U.S.-based residency. Beyond dental school, many graduates choose additional training through General Practice Residencies (GPRs) or Advanced Education in General Dentistry (AEGD) programs, which provide one to two years of advanced, hands-on clinical experience. The profession also includes twelve ADA-recognized specialties, with orthodontics, pediatric dentistry, and oral surgery representing the largest groups. Dental public health remains one of the smallest specialties, with 734 certified specialists, yet plays a critical role in population health and policy. 

Dentists work alongside a large, allied workforce. The country has roughly 220,000 licensed dental hygienists, trained through two- to four-year programs, who focus on preventive services such as cleanings, fluoride treatments, and oral health education. [74]. The scope of practice and supervision requirements for dental hygienists vary across the states [75]. Supervision requirements vary widely; direct supervision in some states (dentist must be on site during the administration of the permitted tasks), indirect supervision (dentist must be on the premises), general supervision (dentist authorizes care but need not be present). Direct access in many states, allowing hygienists to initiate treatment without prior dentist authorization in public health settings. Dental assistants are the largest single group in dentistry, with 381,900 active professionals, most trained through one- to two-year certificates or associate programs [69]. A smaller but emerging role is that of the dental therapist, a mid-level provider trained to deliver a limited range of restorative and preventive services under the supervision of a dentist. Fewer than 150 dental therapists were practicing nationwide as of 2020, concentrated mostly in Alaska, Minnesota, and certain tribal health systems [73]. 

State licensure is mandatory before entering the dental workforce. Dentists must pass national board examinations and a clinical state exam, while hygienists must complete the National Board Dental Hygiene Examination in addition to state requirements. Dental assistants are licensed or registered in some states, reflecting considerable regulatory variation. Dental therapists remain authorized in only a limited number of states—currently Alaska, Minnesota, and Washington. Across all licensed dental professions, continuing education is required to maintain competency and renew licensure. Training programs are also evolving, with growing emphasis on interprofessional collaboration, minimally invasive care, and a stronger focus on population health.

Despite having a relatively large dental workforce overall, the distribution of dental providers is uneven. Urban and suburban areas often have a high concentration of dentists, while many rural and low-income communities experience persistent shortages. The HRSA estimates that approximately 62 million Americans live in designated Dental Health Professional Shortage Areas (HPSAs) [76], nearly two-thirds of which are in rural regions [77]. This imbalance contributes to higher rates of untreated disease and, in some cases, greater reliance on hospital emergency departments for conditions that could have been managed in a dental office. These challenges underscore the need for policies that support a more balanced distribution of providers and expand access to care through workforce innovations. 

According to the 2024 HRSA workforce projections, the U.S. dental labor force is expected to grow steadily but unevenly distributed through 2037 [78]. HRSA’s Health Workforce Simulation Model uses a microsimulation approach to track supply and demand for dentists and dental hygienists, starting from 2022 as the base year. The model adds new graduates each year—approximately 6900 dentists and 7100 dental hygienists—while accounting for retirements, reduced hours with age, and migration between metropolitan and rural areas. Data sources include ADA Master File for dentists and the American Community Survey for hygienists, with service-use patterns estimated from the Medical Expenditure Panel Survey. The projections show that although the national dentist-to-population ratio remains favorable, shortages persist in rural and low-income areas. The model also incorporates a 10% starting shortage for hygienists and assistants, emphasizing the need to expand allied dental education and retention efforts. HRSA evaluated two scenarios: a Status Quo scenario assuming current care patterns continue, and a Reduced Barriers scenario that models higher utilization if historically underserved groups accessed care at the same rate as higher income, insured populations. Under the latter scenario, the demand for full-time equivalent providers would rise substantially, underscoring the importance of geographic redistribution, expanded scope of practice for hygienists, and integration of preventive oral health services into primary care systems. 

In sum, the U.S. dental workforce is large, highly trained, and increasingly diverse, with 79 dental schools and hundreds of residencies and allied training programs supplying new dental professionals each year. At the same time, gaps in distribution and access persist, reminding us that the size of the workforce alone does not guarantee equity. Addressing shortages in underserved areas, expanding training opportunities, and integrating oral health more fully into the broader health system remain essential to ensuring that the benefits of this substantial workforce reach all Americans. 

## 7. Dental Public Health Programs and Preventive Care

A wide range of preventive strategies exists; however, this review focuses on community water fluoridation, school-based dental sealant programs, and topical fluoride treatments, as these interventions are consistently recognized as among the most cost-efficient approaches to reducing dental caries at the population level.

### 7.1. Community Water Fluoridation

Community water fluoridation, introduced in the mid-20th century, is recognized as one of the most effective public health measures in the United States [79]. The practice involves adjusting fluoride levels in public water systems to an optimal concentration of 0.7 milligrams per liter, as recommended by federal health authorities [80]. The extensive scientific literature consistently supports community water fluoridation as a cost-efficient and safe strategy for improving oral health, because it reaches all individuals who rely on municipal water supplies, regardless of socioeconomic status or access to dental care [81]. Currently, about 63% of Americans, or approximately 209 million people nationwide, receive fluoridated water [82]. Coverage varies across states. Hawaii, New Jersey, and Oregon have some of the lowest rates (<30%) of community water fluoridation coverage, while Kentucky, Minnesota, and Illinois rank the highest levels of fluoridated water access (>98%) [80], Economically, the provision of optimally fluoridated water to U.S. communities for a one-year period is estimated to reduce dental treatment expenditures by approximately $6.5 billion, corresponding to an average return on investment of $20 for every $1 invested [83]. Globally, evidence consistently shows that fluoridated water reduces tooth decay by approximately 25% in both children and adults [84]. This reduction translates into estimated savings of roughly $32 per individual per year in avoided treatment costs, in addition to mitigating productivity losses associated with missed work and school days. Beyond cost savings, fluoridation promotes health equity by providing universal access to caries prevention, particularly for underserved populations [85].

However, a recent Cochrane review found that the overall benefits of water fluoridation appear to be less pronounced than in earlier decades, likely due to the widespread availability and use of fluoride toothpaste [84]. Concentrations of fluoride well above those used in community water fluoridation have been found to affect Intelligence Quotient (IQ), a finding that may contribute to the public’s concern about fluoride toxicity even in safe and beneficial concentrations. In August 2024, the U.S. National Toxicology Program released its Monograph on the State of the Science Concerning Fluoride Exposure and Neurodevelopment and Cognition. The report reviewed dozens of studies and concluded that fluoride exposure at levels above the recommended limit (greater than ~1.5 mg/L in drinking water) was associated with lower IQ in children [86]. While the report observes a lack of evidence for cognitive impairment caused by lower fluoride concentrations, there are well-studied effects of higher concentrations on childhood development. Overexposure to fluoride during childhood can cause mild dental fluorosis, which causes white streaks or brown discoloration on developing teeth. At much higher, prolonged levels, it may contribute to skeletal fluorosis, resulting in bone pain and stiffness, though such cases are rare at regulated water concentrations. However, these effects are associated with concentrations far greater than those used for community water fluoridation.

Because of public concern—regardless of its basis in evidence—water fluoridation in the U.S. has faced ongoing challenges. Public opposition, often fueled by misconceptions about health risks, has led to policy reversals in some communities. On September 24, 2024, a California District Court ruled in Food & Water Watch Inc. v. U.S. Environmental Protection Agency [86], Northern District of California) that community water fluoridation may pose a risk of lowering children’s IQ, although harm was not deemed certain [87]. Building on this momentum, at least 21 states introduced legislation in 2025 to prohibit or repeal requirements for adding fluoride to public water supplies, with Utah and Florida becoming the first states to enact this law [88]. While decisions are made at the state or local level, federal agencies provide guidance. Some states mandate fluoridation for large water systems, while others leave the choice to local governments or voters.

Despite these debates, recent meta-analyses confirm that fluoride exposure at levels typical of community water fluoridation does not correlate with reduced IQ in children [89]. Nationwide studies reaffirm that municipal fluoridation does not cause harmful neurodevelopmental effects [90]. A cohort study of more than 11 million births in the U.S. found no link between fluoridated water and adverse birth outcomes, supporting the safety of community water fluoridation during pregnancy [91]. The cost-effectiveness analysis concluded that discontinuing public water fluoridation in the United States would lead to higher rates of tooth decay and increased healthcare expenditures [92]. The American Association for Dental, Oral, and Craniofacial Research (AADOCR) and ADA continue to strongly endorse fluoridation as a safe, cost-effective method of cavity prevention [93,94]. Although there are ongoing debates, legal challenges, and regional variations in coverage, community water fluoridation is supported by decades of rigorous research and a broad scientific consensus affirming its safety, effectiveness, and public health value at recommended levels. In summary, water fluoridation stands as a proven, equitable, and cost-saving measure for reducing dental disease nationwide.

### 7.2. School-Based Dental Sealant Program

Dental caries is the most common chronic disease among children, disproportionately affecting those from low-income families. Molars are especially susceptible to untreated caries from childhood to adulthood [95], highlighting the need for molar-focused preventive strategies. School-based dental sealant programs were developed to address this gap by delivering preventive care directly in schools, particularly to children who may lack regular access to dental services. A systematic review for the U.S. Preventive Services Task Force found that resin-based dental sealants significantly reduce caries risk in first molars (Odds Ratio (OR) = 0.21 at 48–54 months) [96]. Beyond primary prevention, an observational 2-year study in a large dental network concluded that dental sealants applied to occlusal surfaces can arrest noncavitated carious lesions [97].

Evidence consistently demonstrates a strong protective benefit: low-income children without sealants had nearly three times more decayed and filled first molars than those with sealants. It was estimated that 6.5 million children from low-income families could benefit from school-based sealant programs [98]. Results of the CDC-funded initiatives (2013–2018) from 62,750 children in 16 states showed that placing four sealants could prevent decay in one molar. This further highlighted the substantial caries-preventive impact of these programs. Barriers to expanding sealant programs in schools were funding issues and state requirements for on-site dentist supervision during sealant placement and assessment [99].

NHANES data show a 27.5% decline in the measured dental sealant prevalence among children, dropping from 46.6% in 2011–2014 to 33.8% in 2015–2018. Despite this, rates of untreated caries in the first permanent molars of children aged 7–10 years also declined over the same period. Evidence suggests that the lowered measured sealant placement may be due to reduced detection sensitivity—likely due to increased use of glass ionomer sealants, which are more difficult to identify during examinations—rather than an actual reduction in sealant delivery [100]. A cross-sectional study examined the relationship between acculturation and dental sealant use among U.S. children ages 6–18. Fewer than half (45.5%) of the children had sealants overall, and those children living in the U.S. for less than 5 years were significantly less likely to have sealants compared to citizens (22.2% vs. 48.9%; adjusted OR = 0.38). Findings indicate that limited acculturation is associated with lower use of preventive dental services. These results underscore the importance of expanding access to dental sealants for all children—regardless of citizenship status or length of time in the United States—to reduce disparities and lower treatment costs [101].

In addition to their use on permanent teeth, recent research shows that sealants on primary molars also hold significant promise. A cost-effectiveness analysis found that applying resin-based sealants to primary molars in high-risk children under age six is both effective and cost-saving, keeping teeth caries-free longer and reducing treatment costs ($75 vs. $90 per molar). Over a five-year period, this approach could save the U.S. dental system an estimated $742 million, providing strong support for reimbursement policies that include sealant use in young, high-risk populations [102]. However, implementation challenges such as child cooperation and provider-related barriers warrant further investigation.

Currently, the CDC’s Division of Oral Health (DOH) provides funding to strengthen the infrastructure and coordination of school sealant programs. At present, DOH supports initiatives in 15 states and one U.S. territory, enabling the delivery of evidence-based preventive services—particularly in rural schools and those serving low-income students. Program performance is monitored through the Sealant Efficiency Assessment for Locals and States, which enables state and local health officials to assess effectiveness and allocate resources to communities with the greatest need [103].

In sum, expanding the use of dental sealants has the potential to substantially reduce untreated decay and lower overall treatment costs. School-based sealant programs are particularly effective in reducing caries among high-risk children with limited access to dental care. However, addressing persistent policy and funding barriers is essential to support sustainable sealant program expansion and ensure long-term impact.

### 7.3. Fluoride Varnish Delivery Program

Fluoride varnish is widely used in the U.S., though off-label, as a preventive measure against dental caries. Strong evidence shows its effectiveness, reducing caries by approximately 37% in primary teeth and 47% in permanent teeth [104]. National organizations endorse its use: ADA recommends fluoride varnish application for children at elevated risk of tooth decay, AADOCR affirms that topical fluorides, including varnishes at high concentration, are effective for preventing early childhood caries, particularly children under six [105]. The U.S. Preventive Services Task Force recommends primary care clinicians apply fluoride varnish to the teeth of all infants and children starting when primary teeth erupt, particularly for those under age 5 who are at increased risk of tooth decay [106]. Similarly, the American Academy of Pediatrics (AAP) considers fluoride varnish application the standard of care for caries prevention in pediatric primary care [35].

Beyond clinical settings, school-based fluoride varnish programs have demonstrated substantial benefits. A recent systematic review of 31 studies (60,780 students) found that school-based fluoride varnish programs reduce caries initiation by 32% in permanent teeth and 25% in primary teeth, with the strongest benefits among children from low socioeconomic backgrounds [107]. Despite these outcomes, about 30% of U.S. states lack school fluoride varnish programs. The evidence also shows that school-based fluoride varnish programs reduce caries across all school-age groups. It can be implemented in rural and urban communities regardless of fluoridation status and can be administered by both dental and non-dental personnel.

The Community Preventive Services Task Force strongly recommends school-based fluoride varnish delivery programs as an effective strategy to prevent dental caries among children from preschool through high school [108]. Current evidence shows these programs achieve high participation rates, increase the number of fluoride treatments received, reduce cavities in both primary and permanent teeth, and help narrow disparities in oral health outcomes, particularly in low-income communities.

### 7.4. Silver Diamine Fluoride Treatment

Silver diamine fluoride (SDF) is most commonly formulated as a 38% solution containing approximately 44,800 ppm fluoride. SDF has emerged as a minimally invasive, evidence-based intervention for arresting dental caries [109]. Although its use for caries management remains off-label in the United States, SDF is increasingly integrated into clinical practice, particularly for young children under six years of age and populations with limited access to conventional restorative care. ADA also recognizes SDF’s affordability and ease of application, recommending biannual use for caries control, although its U.S. Food and Drug Administration clearance remains limited to treating dentin hypersensitivity [110]. The American Academy of Pediatric Dentistry (AAPD), in its 2024–2025 Reference Manual, conditionally recommends the use of 38% SDF in children and adolescents, including those with special needs, as part of caries management plans [111]. Similarly, AAP issued 2023 guidance for physicians endorsing SDF to arrest decay during routine pediatric visits through age five, supported by the introduction of Current Procedural Terminology code 0792T [111].

Recent studies highlight the growing evidence base for SDF as an effective caries management strategy. A randomized controlled trial demonstrated that a single application of 38% SDF arrested caries in 54% of treated lesions at six months, compared to only 21% in the placebo group, leading to early trial termination due to significant efficacy [112]. Similarly, the CariedAway school-based cluster randomized trial reported that SDF combined with fluoride varnish achieved outcomes comparable to traditional sealants plus varnish, with approximately 56% caries arrest and 81% prevention over a two-year period [113]. These findings underscore SDF’s potential as a minimally invasive alternative for caries management in community and school-based programs. Educational adoption further reflects this trend. According to a 2025 national survey, all U.S. dental schools now include SDF in their curricula, compared with only 68% in 2016 [114]. This integration ensures that future practitioners are equipped to implement evidence-based, non-invasive caries management strategies.

In summary, community water fluoridation, school-based dental sealants, and topical fluoride applications constitute cornerstone strategies in caries prevention. These interventions are widely recognized as cost-effective and scalable, offering substantial benefits at the population level. More importantly, they extend preventive protection to groups with limited access to conventional dental care. Nevertheless, their implementation is often hindered by political resistance, resource constraints, and inequitable access. Addressing these barriers requires sustained policy advocacy, enhanced public education, and strategic investment in workforce development and infrastructure.

## 8. Challenges and Opportunities

Despite overall improvements in national oral health since the 1960s, vulnerable population groups—including low-income families, racial and ethnic minorities, and rural residents—experience disproportionately high levels of untreated disease due to structural barriers that limit access to preventive and restorative services [115]. Racial and ethnic disparities remain pervasive, with Black Americans experiencing a disproportionately higher burden of oral disease across most measured outcomes [116]. Non-Hispanic white children were more likely to receive fluoride treatments and dental sealants than minority groups while Asian American children showed a significant decline in sealant use and were more likely to have dental caries than non-Hispanic white children [117]. No major improvements in disparities were observed over time, underscoring the need for continued efforts to expand access to preventive dental services for minority and low-income populations [117].

Tooth disorders accounted for an annual average of 1.94 million emergency department visits between 2020–2022. Surprisingly, adults aged 25–34 represented the largest proportion of these visits (29.2%) and Medicaid was the primary expected source of payment for most visits (55%) [118]. Social determinants of health substantially influence dental access to care services. Lack of health insurance, annual income below $35,000, and housing instability were each significantly associated with unmet dental care needs [119]. Interestingly, these disparities were most pronounced among young adults (18–35 years), a group often assumed to have better health and access to care. However, their unique vulnerabilities—such as unstable employment, lack of insurance, student debt, and housing insecurity—are frequently under-recognized compared to older adults or children. These findings underscore the expansion of affordable coverage and reduction of cost barriers by extending comprehensive adult dental benefits in Medicaid, including for low-income adults. Affordability remains a major obstacle: dental care is consistently reported as one of the most costs-prohibitive types of healthcare.

According to the CareQuest Institute’s latest State of Oral Health Equity in America survey, about 72 million U.S. adults (27%) lack dental insurance—nearly three times the proportion of adults without medical insurance [120]. This burden is more pronounced among seniors. Advances in research and preventive care have improved outcomes; however, many Americans still lack affordable access to these services. As of late 2025, approximately 62 million people live in areas with shortages of dental providers, and most of these shortage areas are in rural communities [77]. Approximately 10,400 additional dental professionals are required to eliminate these shortages. Additionally, oral health remains largely siloed from the broader medical care system, limiting opportunities for integration and coordinated prevention. Systemic barriers—such as wide variation in Medicaid adult dental benefits across states and restrictive scope-of-practice regulations—further constrain the ability of dental hygienists and other mid-level providers to expand access in underserved communities. In summary, key challenges facing the U.S. oral health system are summarized as follows; (1) persistent inequities in access, (2) financial barriers, (3) limited access in rural or underserved areas, (4) workforce distribution and shortage, and (5) a fragmented dental care delivery system.

Together, these challenges underscore the need for strategic and evidence-based solutions. Several key opportunities or calls to action, which can strengthen the U.S. oral health system, are detailed below.

### 8.1. Expand Evidence-Based and Cost-Effective Prevention to High-Risk Populations

Scaling up proven preventive interventions—such as school- and community-based sealant programs, fluoride varnish, SDF, and community water fluoridation—offers one of the most efficient pathways to reducing disease burden and long-term costs. Prioritizing high-risk populations is especially important. A simulation of 50,000 U.S. children found that risk-based prevention was more cost-effective, demonstrating that targeting high-risk children yields economic value [121].

### 8.2. Strengthen Medicaid and Public Insurance Coverage

Expanding comprehensive adult dental benefits in Medicaid—particularly for disadvantaged populations—and improving provider reimbursement and administrative processes would substantially increase participation and access. Enhanced coverage and reimbursement can reduce financial barriers and narrow persistent oral health disparities.

### 8.3. Integrate Oral Health into Primary Care

Embedding oral health screening, fluoride varnish, SDF for caries control, anticipatory guidance, and referral pathways into pediatric and adult primary care can facilitate early detection and reduce avoidable emergency department visits. Medical–dental integration models offer a practical mechanism for reaching patients who may not otherwise access dental services.

### 8.4. Expand and Diversify the Oral Health Workforce

Addressing workforce shortages requires supporting dental therapists, expanded-function dental hygienists, and community health workers, and updating scope-of-practice regulations to allow dental-auxiliary providers to practice at the highest level of their competencies. In addition, increasing workforce diversity and expanding positions for dental public health specialists who can lead advocacy, research, and community partnerships are essential for improving access and advancing equity.

### 8.5. Leverage Teledentistry and Digital Innovation

Teledentistry offers a scalable tool for expanding access through virtual triage, remote monitoring, and mobile care delivery. Evidence shows that hygienist-led and asynchronous teledentistry models effectively reach schools, rural clinics, and long-term care facilities, reducing travel burdens and improving referral pathways. Digital tools such as mobile health applications, AI-supported risk assessment, and remote monitoring devices further enhance patient engagement and early detection of disease. Electronic health record integration and interoperable data systems also strengthen medical–dental collaboration, enabling primary care teams to identify oral health needs and connect patients to appropriate services [122,123].

### 8.6. Address Social and Structural Determinants of Oral Health

Policy actions that improve access to healthy foods, reduce sugar consumption, and strengthen public health protections can mitigate upstream drivers of disease. Expanded government-funded dental services can also reduce the impact of structural inequities on oral health, particularly for vulnerable populations facing compounded social determinants. Many individuals continue to forgo treatment due to cost, highlighting the need for broader coverage reforms, and continued investment in community-based prevention.

### 8.7. Advance Health Equity Through Policy and Research

Improving oral health equity requires system change, including policy reform and community-engaged research. The practices of using data to identify disparities, investing in culturally responsive care, and supporting community-driven solutions are essential. Given the substantial economic burden of oral diseases, including more than $200 billion in productivity losses, shifting from treatment to prevention through integrated policy and equitable financing models offers a long-term impact. Public health perspectives are central to advancing interdisciplinary research and ensuring that scientific advances translate into population-level improvements [124].

Although this review presents an overview landscape of oral health care and highlights key challenges and opportunities in improving oral health in the United States, several limitations should be acknowledged. First, as a narrative review, the selection of studies may be influenced by publication availability and author judgment, which introduces the potential for selection bias. Second, given the breadth of this review, it was not feasible to capture all studies relevant to each theme. Spatial limitations necessitated prioritizing high-quality, illustrative evidence, which may omit additional studies that could further enrich the discussion. Finally, the rapidly evolving landscape of oral health policy and reporting systems means that some findings may shift over time, and newer developments may not be fully reflected in this synthesis. These limitations underscore the need for ongoing systematic evidence reviews to guide practice and research priorities.

Future research should examine scalable preventive strategies such as school-based and community-based programs using evidence-based interventions to determine how they can be more effectively implemented in underserved and rural regions. Additional studies are needed to evaluate integrated medical–dental care models, particularly in areas facing persistent workforce dental shortages and limited public coverage. Finally, rigorous evaluation of innovative workforce models, including expanded roles for mid-level dental providers, is essential to establish their potential to reduce the national burden of oral disease.

## 9. Conclusions

Oral diseases remain widespread in the U.S. Despite advances in prevention and treatment, socioeconomic and geographic disparities continue to restrict equitable access to dental care. Closing these gaps will require integrating oral health into the broader health system, expanding affordable insurance coverage, and strengthening prevention at the population level. Prioritizing these efforts is essential to improving oral health for all Americans.

## Figures and Tables

**Table 1:** Prevalence and severity of dental caries, and retained teeth and edentulism across age groups, based on the (NHANES) 2017–2020 prepandemic [10,11].

Age Group (Years)	Prevalence of Untreated Decay (%) ^3^	Mean Decayed Teeth (dt/DT) ^4^	Mean Filled Teeth (ft/FT) ^4^	Mean Number of Retained Teeth	% Edentulous ^5^
2–5 ^1^	11.1	1.8	2.6	–	–
6–8 ^1^	17.7	0.9	3.2	–	–
6–11 ^2^	2.6	0.3	1.6	–	–
12–19 ^2^	10.4	0.4	3.7	–	–
20–34 ^2^	21.8	0.8	4.8	27.0	–
35–49 ^2^	21.4	0.6	6.0	25.6	1.2
50–64 ^2^	17.3	0.4	7.5	23.3	5.9
65–74 ^2^	12.4	0.2	9.0	21.7	11.4
≥75 ^2^	12.6	0.2	9.7	19.8	19.7

^1^ Data refer to primary teeth, ^2^ Data refer to permanent teeth, ^3^ Untreated decay prevalence = % with ≥1 untreated decayed tooth; for adults (≥20), estimates are among dentate participants (≥1 natural tooth), ^4^ Mean decayed teeth (dt/DT) and mean filled teeth (ft/FT) are reported among participants with caries experience (dft ≥ 1 for primary teeth; DMFT ≥ 1 for permanent teeth) for ages <65; for ages ≥65, these means are reported among dentate adults (≥1 natural tooth), ^5^ Edentulism = complete loss of natural teeth.

**Table 2:** Prevalence and severity of dental caries, and retained teeth and edentulism across age groups, based on the (NHANES) 2011–2016 [12].

Age Group (Years)	Prevalence of Untreated Decay (%) ^3^	Mean Decayed Teeth (dt/DT) ^4^	Mean Filled Teeth (ft/FT) ^4^	Mean Number of Retained Teeth	% Edentulous ^5^
2–5 ^1^	10.4	1.6	2.8	–	–
6–8 ^1^	16.4	0.8	3.6	–	–
6–11 ^2^	5.2	0.5	1.6	–	–
12–19 ^2^	16.6	0.7	3.4	–	–
20–34 ^2^	29.3	1.2	4.9	27.0	–
35–49 ^2^	26.4	0.9	6.6	25.5	1.6
50–64 ^2^	21.5	0.6	8.3	23.4	5.6
65–74 ^2^	15.4	0.3	9.9	21.7	13.0
≥75 ^2^	16.5	0.3	9.6	19.5	22.5

^1^ Data refer to primary teeth, ^2^ Data refer to permanent teeth, ^3^ Untreated decay prevalence = % with ≥1 untreated decayed tooth; for adults (≥20), estimates are among dentate participants (≥1 natural tooth), ^4^ Mean decayed teeth (dt/DT) and mean filled teeth (ft/FT) are reported among participants with caries experience (dft ≥ 1 for primary teeth; DMFT ≥ 1 for permanent teeth) for ages <65; for ages ≥65, these means are reported among dentate adults (≥1 natural tooth), ^5^ Edentulism = complete loss of natural teeth.

**Table 3:** Oral cavity and pharynx cancer incidence rates (per 100,000), U.S., in 2015–2019 [21,22,23].

Group/Age	Incidence Rate ^1^ (per 100,000)
All ages	11.5
<15 years	0.1
15–39 years	1.4
40–64 years	17.8
65–74 years	43.7
75+ years	44.8

^1^ Rates are age-adjusted to the 2000 U.S. standard population.

**Table 4:** Sources of Dental Expenditures in the U.S.

Payment Source	Share of Total Dental Expenditure	Notes
Out-of-pocket	40%	Patients are responsible for a portion of the cost of preventive, restorative, and prosthodontic services, reflecting limited insurance coverage and annual caps
Private dental insurance	35%	Employer-sponsored and individual plans; emphasis on preventive care; annual maximums typically US$1000–1500
Medicaid	12%	Primary public insurance program for low-income individuals and those with special needs. Dental benefits are mandatory for children and optional for adults, with coverage varying by state
Children’s Health Insurance Program (CHIP)	3%	Provides comprehensive health and dental benefits for low- to moderate-income children
Medicare	1%	Covers only medically necessary dental services linked to medical care; it has accounted for most of the recent increase in dental spending
Other public insurance programs	2%	Includes Veterans Health Administration, military programs, and other federal systems.

Note: Data compiled from ADA Health Policy Institute [46,50].

**Table 5:** Number of dental institutions and registered dental professionals in each dental degree (Updated on 30 September 2025 [69,70,71].

Dental Degree (Duration of Study)	Number of Schools/ Institutions [71]	Number of Registered Dental Professionals [8]
**Predoctoral:** Doctor of Dental Surgery (DDS) or Doctor of Dental Medicine (DMD) degree (3–4 years after undergraduate education)	79	202,485
**Postgraduate residency programs**		
General Practice & Primary Care		
General Practice Residency (GPR)—hospital-based, broad clinical training. (12 or 24 months)	167	No data available
Advanced Education in General Dentistry (AEGD)—practice-based, focus on comprehensive care. (12 or 24 months)	97	No data available
ADA-Recognized Dental Specialties (2–3 years)		
Dental Anesthesiology	9	416
Dental Public Health	16	734
Endodontics	56	5471
Oral and Maxillofacial Pathology	15	428
Oral and Maxillofacial Radiology	9	738
Oral and Maxillofacial Surgery	103	7424
Oral Medicine	6	521 [72]
Orofacial Pain	14	958
Orthodontics and Dentofacial Orthopedics	69	10,830
Pediatric Dentistry	89	9312
Periodontics	57	5685
Prosthodontics	48	3540
**Dental Therapist:** Bachelor’s degree (2–4 years)	3	<150 (reported in 2020) [73]
**Dental Hygiene**: Associate or bachelor’s degree (2–4 years)	345	220,000 [74]
**Dental Assisting:** Certificate or associate degree (1–2 years)	223	381,900 [69]

## Data Availability

As the data used in this study were generated from published sources, all information is publicly available.

## Abbreviations

The following abbreviations are used in this manuscript:

AADOCR	American Association for Dental, Oral, and Craniofacial Research
AAP	American Academy of Pediatrics
AAPD	American Academy of Pediatric Dentistry
ADA	American Dental Association
AEGD	Advanced Education in General Dentistry
CDC	Centers for Disease Control and Prevention
CHC	Community Health Center
CHCS	Center for Health Care Strategies
CHIP	Children’s Health Insurance Program
CMS	Centers for Medicare & Medicaid Services
DOH	Department of Health
DDS	Doctor of Dental Surgery
DMD	Doctor of Dental Medicine
GDP	Gross Domestic Product
GPR	General Practice Residency
HPV	Human Papillomavirus
HPSA	Health Professional shortage Area
HRSA	Health Resources and Services Administration
IQ	Intelligence Quotient
Medicaid	Medical Assistance Program (U.S. public insurance for low-income individuals)
Medicare	U.S. federal health insurance program for adults ≥65 years and certain disabilities
NHANES	National Health and Nutrition Examination Survey
OR	Odds Ratio
SDF	Silver Diamine Fluoride
U.S.	United States
US$	United States Dollar
